# Early to late sparing of radiation damage to the parotid gland by adrenergic and muscarinic receptor agonists

**DOI:** 10.1054/bjoc.2001.2038

**Published:** 2001-09

**Authors:** R P Coppes, L J W Zeilstra, H H Kampinga, A W T Konings

**Affiliations:** Department of Radiation and Stress Cell Biology, University of Groningen, Ant. Deusinglaan 1, Groningen, AV, 9713, The Netherlands

**Keywords:** head and neck cancer, irradiation, prophylactic treatment, parotid gland

## Abstract

Damage to salivary glands after radiotherapeutic treatment of head and neck tumours can severely impair the quality of life of the patients. In the current study we have investigated the early-to-late pathogenesis of the parotid gland after radiation. Also the ability to ameliorate the damage using pretreatment with adrenergic or muscarinic receptor agonists is studied. Rats were locally irradiated with or without i.p. pretreatment with phenylephrine (α-adrenoceptor agonist, 5 mg kg^−1^), isoproterenol (β-adrenoceptor agonist, 5 mg kg^−1^), pilocarpine (4 mg kg^−1^), methacholine (3.75 mg kg^−1^) (muscarinic receptor agonists) or methacholine plus phenylephrine. Parotid salivary flow rate, amylase secretion, the number of cells and gland histology were monitored sequentially up to 240 days postirradiation. The effects were described in 4 distinct phases. The first phase (0–10 days) was characterised by a rapid decline in flow rate without changes in amylase secretion or acinar cell number. The second phase (10–60 days) consists of a decrease in amylase secretion and is paralleled by acinar cell loss. Flow rate, amylase secretion and acinar cell numbers do not change in the third phase (60–120 days). The fourth phase (120–240 days) is determined by a further deterioration of gland function but an increase in acinar cell number, albeit with poor tissue morphology. All drug pretreatments used could reduce radiation effects in phase I and II. The protective effects were lost during phase IV, with the exception of methacholine plus phenylephrine pretreatment. The latter combination of drugs ameliorated radiation-damage throughout the entire follow-up time. The data show that combined pre-irradiation stimulation of muscarinic acetylcholine receptors with methacholine plus α-adrenoceptors with phenylephrine can reduce both early and late damage, possibly involving the PLC/PIP2 second messenger pathways. This opens perspectives for the development of clinical applicable methods for long-term sparing of parotid glands subjected to radiotherapy of head and neck cancer patients. © 2001 Cancer Research Campaignhttp://www.bjcancer.com


*British Journal of Cancer* (2001) **85**(7), 1055–1063
© 2001 Cancer Research Campaign
doi: 10.1054/ bjoc.2001.2038, available online at http://www.idealibrary.com on IDEAL

http://www.bjcancer.com

### Early to late sparing of radiation damage to the parotid gland by adrenergic and muscarinic receptor agonists

**RP Coppes, LJW Zeilstra, HH Kampinga and AWT Konings**

Department of Radiation and Stress Cell Biology, University of Groningen, Ant. Deusinglaan 1, 9713 AV Groningen, The Netherlands

**Summary** Damage to salivary glands after radiotherapeutic treatment of head and neck tumours can severely impair the quality of life of the patients. In the current study we have investigated the early-to-late pathogenesis of the parotid gland after radiation. Also the ability to ameliorate the damage using pretreatment with adrenergic or muscarinic receptor agonists is studied. Rats were locally irradiated with or without i.p. pretreatment with phenylephrine (α-adrenoceptor agonist, 5 mg kg$^{-1}$), isoproterenol (β-adrenoceptor agonist, 5 mg kg$^{-1}$), pilocarpine (4 mg kg$^{-1}$), methacholine (3.75 mg kg$^{-1}$) (muscarinic receptor agonists) or methacholine plus phenylephrine. Parotid salivary flow rate, amylase secretion, the number of cells and gland histology were monitored sequentially up to 240 days postirradiation. The effects were described in 4 distinct phases. The first phase (0–10 days) was characterised by a rapid decline in flow rate without changes in amylase secretion or acinar cell number. The second phase (10–60 days) consists of a decrease in amylase secretion and is paralleled by acinar cell loss. Flow rate, amylase secretion and acinar cell numbers do not change in the third phase (60–120 days). The fourth phase (120–240 days) is determined by a further deterioration of gland function but an increase in acinar cell number, albeit with poor tissue morphology. All drug pretreatments used could reduce radiation effects in phase I and II. The protective effects were lost during phase IV, with the exception of methacholine plus phenylephrine pretreatment. The latter combination of drugs ameliorated radiation-damage throughout the entire follow-up time. The data show that combined pre-irradiation stimulation of muscarinic acetylcholine receptors with methacholine plus α-adrenoceptors with phenylephrine can reduce both early and late damage, possibly involving the PLC/PIP2 second messenger pathways. This opens perspectives for the development of clinical applicable methods for long-term sparing of parotid glands subjected to radiotherapy of head and neck cancer patients. © 2001 Cancer Research Campaign http://www.bjcancer.com

**Keywords**: head and neck cancer; irradiation; prophylactic treatment; parotid gland

The salivary glands are tissues at risk during radiotherapeutic treatment of head and neck tumours. Exposing the salivary glands to radiation often results in a progressive irreversible loss of gland function, which may persist during the rest of the patient's life, leading to irreversible and distressing oral complaints (Shannon et al, 1978; Valdez et al, 1992; Leslie and Dische, 1994). These consequences of the treatment have a negative impact on the quality of life of the patients (Jensen et al, 1994). Therefore protection of the salivary glands against radiation damage is of great importance.

The loss of salivary flow may already be observed after a few fractions of radiation (Shannon et al, 1978). This early response on gland function has been studied thoroughly in rats (e.g. Vissink et al, 1990; Franzén et al, 1991; Nagler et al, 1993; Coppes et al, 1997a, b). Within 3 days after irradiation with a single dose of 15 Gy of X-rays a decrease in salivary flow rate of near 50% can be observed (Vissink et al, 1990; Peter et al, 1995; Coppes et al, 1997a, b). The literature on radiation-induced changes in morphology is somewhat contradictory. Observations regarding loss of gland weight and acinar cells (Phillips, 1970; Sodicoff et al, 1974), the latter being the major sites of fluid production (Garrett and Proctor, 1998), stand in contrast to the lack of obvious quantitative morphological alterations (Franzén et al, 1991; Henricksson et al, 1994) and the lack of increase in apoptotic cells (Paardekooper et al, 1998) early after irradiation. The early effects of radiation on the parotid gland may therefore rather be due to dysfunctioning of non-removed cells at the level of membranes (Sodicoff et al, 1974; EI Mofty and Kahn, 1981; Vissink et al, 1992) and/or intracellular signalling (Vissink et al, 1991; Coppes et al, 1997b). The late effects of radiation on the parotid gland have been studied less extensively, but can be described by a dose-dependent further decline in function (Nagler et al, 1998), loss of acinar cells and the development of fibrosis (Henricksson et al, 1994). Regretfully, in the latter studies the whole or half of the head including the glands were irradiated. Therefore indirect effects due to damage to other organs confounds the interpretation with regards to salivary gland function. No study so far has been performed studying the early-to-late effects of radiation on parotid gland function in the same rat. The approach used in this study provides the means to determine, for the first time, changes in parotid gland function over a longer period within the same rat.

Xerostomia is multifactorial depending on changes in salivary output from all major glands as well as the minor glands (Eisbruch et al, 1999). Although parotid gland dysfunction does not necessarily lead to xerostomia, it is of great clinical importance to find drugs that are able to protect against radiation damage to the glands, especially against late effects. Pilocarpine has been used to relieve the symptoms of postirradiation xerostomia (Johnson et al, 1993; Leveque et al, 1993). Recently, prophylactic treatments with pilocarpine and some related drugs have been shown (Peter et al,

*Received 29 September 2000*
*Revised 14 June 2001*
*Accepted 20 June 2001*

*Correspondence to:* RP Coppes

1995; Coppes et al, 1997a, b) to protect against the early (0–30 days post irradiation) effects of radiation on rat salivary gland function.

Since nothing is known about the duration of the protection, these studies were extended till 240 days after irradiation in the current study. For this purpose, we locally irradiated the parotid glands of the rat with or without pretreatment with adrenergic and muscarinic receptor agonists consecutively following early-to-late radiation damage. A single dose of 15 Gy was choosen as earlier studies (Vissink et al, 1990, Roesink et al, 1999) have shown that a saturation of the radiation effects occurs at higher doses. Whereas rapid function loss after irradiation was characterised by a rapid decline in flow rate without changes in amylase secretion or acinar cell number, amylase secretion decreased later after radiation in parallel with a loss of acinar cells. Very late after radiation, the gland function further deteriorated despite an increase in acinar cell number that, however, was associated with a poor overall tissue morphology with some signs of fibrosis.

We found that whereas pretreatment with most drugs can only temporarily ameliorate gland function after X-rays, a combination of the muscarinic receptor agonist methacholine plus the α-adrenergic receptor agonist phenylephrine was effective over the whole follow-up period.

Information obtained by parallel detection of gland function (flow rate, amylase secretion), general morphology, cell percentages and gland weight allowed us to postulate a mechanism of the sequential radiation-induced damage to the parotid gland and protection against this damage. The latter provides us with tools to design clinically applicable protocols to ameliorate the short- and long-term side effects of radiotherapeutic treatment of head and neck cancer.

### MATERIALS AND METHODS

### Animals

Young adult (8–9 week old) male albino Wistar rats (strain Hds/Cpb: WU), weighing approximately 230 g, purchased from Harlan CPB Rijswijk, The Netherlands, were used in all studies. They were kept in polycarbonate cages (6 rats per cage) under a 14:10-h light : dark cycle. The rats were housed for 10 days prior to the experiments. Food (RMH-B, Hope Farms, Woerden, the Netherlands) and water was given ad libitum. All experiments were performed in agreement with the Netherlands Experiments on Animal Act (1977), the European Convention for the Protection of Vertebrates Used for Experimental Purposes (Strasbourg, 18.III.1986) and met the standards required by the UKCCCR Guidelines (UKCCR, 1998).

### Parotid gland irradiation

The rats were irradiated as previously described (Coppes et al, 1997a). Prior to irradiation all rats were anaesthetised by an i.p. injection of Ketamine (Ketalar, 60 mg $kg^{-1}$) and Xylazine (Rompun, 2.5 mg $kg^{-1}$). A 6 mm lead shield with a portal of $2 \times 5$ cm was positioned over the body of the rat so that, except for the parotid/submandibular region, the body, including the oral cavity, was excluded from the irradiation field (Coppes et al, 1997a). Both glands were irradiated with a single dose of 15 Gy. The X-ray apparatus (Mueller MG 300, Philips, Eindhoven, The Netherlands) was operated at 15 mA, 200 kV (filters: 0.5 mm copper and 0.5 mm aluminium; HVL = 1 mm Cu). The treatment distance to the focal spot of the skin was 32.5 cm, leading to a dose rate at the gland level of 1.0 Gy $min^{-1}$. This dose rate was determined in air with a calibrated electrometer and ionisation chamber combination (Keithley 35040+NE-2571). The tissue outside the primary beam received less than 1% of the dose applied.

### Treatments

The animals were divided randomly into 7 groups. The sham group was sham-irradiated and sham-treated (1 ml $kg^{-1}$ saline, 1 h prior to sham-irradiation). The control group was sham-treated prior to irradiation. The other groups were treated before irradiation, with adrenergic and/or muscarinic acetylcholine receptor (MAChR) agonists. One group was treated with the α-adrenergic receptor (α-AR) agonist phenylephrine, 5 mg $kg^{-1}$, 60 min prior to irradiation. A second group was treated with the β-adrenergic receptor (β-AR) agonist isoproterenol 5 mg $kg^{-1}$, 90 min prior to irradiation. A third group was treated with methacholine 3.75 mg $kg^{-1}$, 60 min prior to irradiation. A fourth group was treated with pilocarpine, 4 mg $kg^{-1}$, 60 min prior to irradiation and a fifth group was treated with methacholine, 3.75 mg $kg^{-1}$ plus phenylephrine, 5 mg $kg^{-1}$, 120 and 60 min prior to irradiation, respectively. The selection of drugs, drug combinations as well as most dose-schedules originate from the studies to test the degranulation concept (Coppes et al, 1997a, b). In these studies, we found short-term protection of both non-degranulating and degranulating drug pre-treatments. The dose schedules were based on obtaining maximal receptor stimulation.

### Drugs

Phenylephrine (L-phenylephrine-hydrochloridium, Sigma, St. Louis, MO), Isoproterenol (dl-isoproterenol-hydrochloride, Sigma), Methacholine (acetyl-β-methylcholine chloride, Aldrich Chemical Co., Milwaukee) and Pilocarpine, ((+)-pilocarpine hydrochloride, University Pharmacy, Groningen, The Netherlands), were dissolved in saline and injected i.p.

### Collection of saliva

For saliva collection experiments in total 56 animals were used, 8 per experimental group. All collections were performed under anaesthesia. The rats were randomly divided in time for radiation and sampling. The time between irradiation and the first sampling at day 1 was between 22–24 hours. The sham rats were also anaesthetised to excluded anaesthesia effects on food and water intake and (subsequent) saliva flow. The rats were anaesthetised by an i.p. injection of 60 mg $kg^{-1}$ Brietal® and intubated to minimise respiratory complications. Saliva samples of both left and right parotid gland were collected at the same time under halothane/$N_2O/O_2$ anaesthesia by means of miniaturised Lashley cups (Vissink et al, 1989). The cups were placed upon the orifices of both parotid glands. Saliva was collected for 30 min after stimulation with 2 mg $kg^{-1}$ pilocarpine administered subcutaneously (given at t = 0 and t = 15 min). Parotid saliva secretion was collected in preweighted ice-cooled plastic tubes. Saliva was collected 4 days before and 1, 3, 7, 10, 30, 60, 120, 180 and 240 days after irradiation. As a parameter for the assessment of parotid gland function, saliva flow rates and amylase secretion were determined. The total volume of saliva secreted was estimated by

 *© 2001 Cancer Research Campaign*

weight assuming the specific gravity of saliva of 1.0 g cm$^{-3}$. The salivary flow rate (µl min$^{-1}$) was calculated from the collecting time (min) and volume of saliva secreted (µl). Amylase secretion was determined by multiplying the amylase concentration in the collected saliva as quantified by the method of Pierre and Nadj (1976) by the flow rate. The flow rate and amylase secretion was expressed as % of the value before irradiation (± SEM).

### Morphology

For histopathology 3 rats were sacrificed for each time point 1, 3, 7, 10, 30, 60, 120, 180 and 240 days after treatment with radiation alone (27 rats) or after different drug pre-treatment (phenylephrine, pilocarpine, methacholine and methacholine plus phenylephrine) followed by radiation (108 rats). As a control for age-dependent changes, gland histology was assessed in sham-treated rats 4 days before, 10, 30, 120, 180 and 240 days (18 rats). The rats were anaesthetised by an i.p. injection of 60 mg kg$^{-1}$ sodium pentobarbital (University Pharmacy, Groningen, The Netherlands). The animals were exsanguinated before the parotid glands were prepared free from surrounding tissue. Hereafter the glands were extirpated. After rinsing with PBS the wet weight of the glands was determined and subsequently the tissue was incubated for 24 h at room temperature in a 5% (v/v) solution of formaldehyde in phosphate buffer, pH 7.4. Following dehydration using graded alcohol series, the tissue was embedded in Technovit 7100 resin (Heraeus Kulzer GmbH, Wehrheim, Germany) and using a microtome (Jung, Heidelberg, Germany), 2 µm sections were cut. The sections were stained with Haematoxylin (Gill No 2, Sigma diagnostics, St Louis, MO) and eosin (bluish eosin, Chroma-Gesellschaft, Koengen, Germany). The sections were coded and scored in a random order by a certified histologist (LJWZ), who was blinded for all sections including the treatment arm. Internal controls for the observer variability were included. Acinar, intercalated duct and striated duct cells were counted in 5 randomly chosen fields per section (field size: 145 × 215 µm$^2$ at 600 × magnification). Aberrant nuclei, vacuoles, granulation level of acinar cells, cell–cell contact, contact between acini, blood vessels and connective tissue were scored arbitrary by comparing them to control and non-pretreated irradiated glands. The number of cells scored in 5 randomly chosen high-power fields per section were averaged. This value was multiplied by the gland weight of that rat divided by the average gland weight of unirradiated rats 4 days before treatment. Thus a value in arbitrary units proportional to the absolute number of cells of that type per gland was obtained. The number of cells calculated for sham-treated 4 days before treatment was set to 100%. The values for the other conditions were then expressed as a percentage (± SEM) of this control.

### Data analysis

The results are described in intervals. Acute, early, intermediate and late effects describe the events happening during the first 10 days, 10–60 days, 60–120 days and 120–240 days after irradiation, respectively. The choice of the intervals is, of course, also dependent on the available points of measurement and therefore necessarily global. As a result also the periods in time that different forms of damages become manifest, are rough estimations.

The results were analysed using a 2-sided Student's *t*-test for paired or unpaired data; probabilities below 5% were taken to be significant.

### RESULTS

### Adverse effects

No radiation-induced mortality was observed. Out of a total of 56 rats used in the saliva collection experiments, 15 died before the end of the experimental period during the recovery from anaesthesia. This was due to saliva leakage into the bronchus after removal of the Lashley cups. This occurred without any relation to the treatment the rats received. This means, however, that with time after treatments the number of measurements per treatment group was reduced. However, the variation between the data within the same group at a certain time point was small enough to reveal significant inter-treatment differences even at the later time-points with reduced numbers of animals. At the end of the experiments at least 5 rats per experimental group remained. None of the rats used for morphology died prematurely. Development of pseudomembranes or ulcers, known to interfere with the nutritional status, was observed in none of the cases. At 60 days after irradiation some loss of hair was observed in the irradiated groups at the position of the radiation portal. The animals weighed 280 ± 3 g at the start of the experiment. The body weight of all irradiated rats decreased reaching a minimum 6 days after irradiation (about 93% of the original body weight). Examination of the oral cavity did not reveal any signs of mucositis during the first week of sampling. From day 10 after irradiation all body weights increased in parallel with the shams, never completely making up for the difference. All drugs and combinations of drugs were also tested in the non-irradiated situation. The drug treatment as such, did not result in changes in flow rate, amylase secretion or cell numbers (see Coppes et al, 1997a, b). Drug induced changes in mean arterial blood pressure and heart rate, as measured by the tail-cuff method, were returned to (near) normal at the time of radiation.

Up to now, no data are available which in consecution describe acute-to-late irradiation-induced changes in parotid gland function and morphology. To establish the effect of 15 Gy of X-rays, successive measurements of flow rate, amylase secretion, morphology and number of acinar cells/gland were performed up to 240 days after irradiation. The results obtained are plotted in Figure 1, expressed as percentage of values before irradiation. Prior to irradiation the mean flow rate of all rats was 10.1 ± 0.3 µl min$^{-1}$, the amylase secretion was 78.3 ± 4.9 U min$^{-1}$, number of acinar cells per high power field was 4.9 ± 0.2 cells µm$^{-2}$, and the gland weight was 206 ± 14 mg.

### Effects of irradiation on flow rate, amylase secretion, acinar cell number and tissue morphology

Flow rate was determined to quantify the capability of the gland to secrete saliva. As shown in Figure 1, radiation caused a decline in pilocarpine-induced saliva flow rate. Firstly, a rapid decline is observed with a minimum already reached at 6 days postirradiation (Figure 1A). Secondly, a significant further deterioration between 180 and 240 days was found, leading to a secretory level of 31 ± 5% ($P < 0.01$) of the control (Figure 1B).

Amylase secretion was determined to evaluate the quality of the saliva. Similar to the changes in flow rate, radiation induced a 2-phased reduction in amylase secretion, but with different kinetics. No significant decrease in amylase secretion up to 10 days after irradiation was observed (Figure 1A). This is consistent with earlier reports (Gustafsson et al, 1992; Peter et al, 1995) but

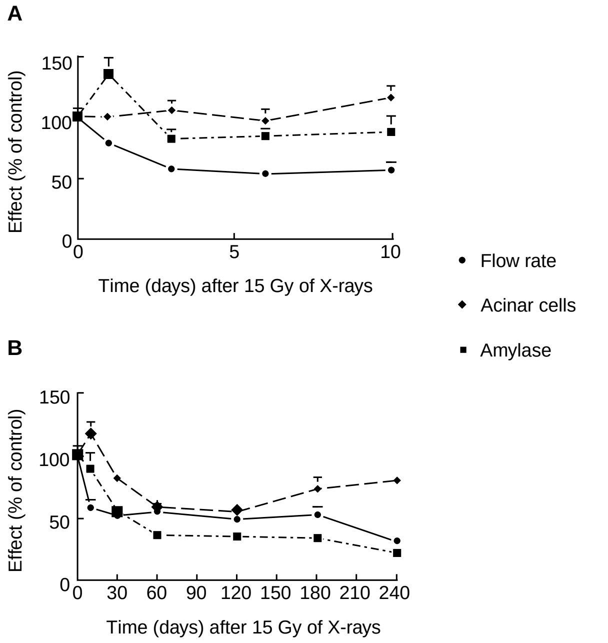

**Figure 1** Changes in parotid gland flow rate (circles), amylase secretion (squares) and acinar cell number (diamonds) following local irradiation with 15 Gy of X-rays, as a function of time after treatment. The number of acinar cells are calculated as the number of cells counted multiplied by the ratio of the gland weight of the experimental rat and the average gland weight of age-matched control rats. Data are expressed as a percentage of pre-treatment control values and are the mean values (± SEM)

contrasts data by Nagler et al (1993). However, in the latter study, radiation effects on amylase secretion may have been caused by an oropharyngeal syndrome in the animals due to irradiation of the entire head. The major decline in amylase secretion started between 10 and 30 days after irradiation reaching a minimum after 60 days (Figure 1B). A second less pronounced decline occurred, more or less parallel to the flow rate, between 180 (range 17.9–48.9%) and 240 (range 2.8–32.8%) days postirradiation ($P < 0.05$).

Radiation did not induce changes in acinar cell number up to 10 days postirradiation (Figure 1A). From day 10 on, the acinar cell number decreased in parallel with the amylase secretion (Figure 1B). Immediately after irradiation a decrease in granulation level and an increase in size and number of vacuoles of the acinar cells was observed. The number of aberrant nuclei increased but never exceeded a level of 3% (see also Paardekoper et al, 1998). The tissue morphology of the gland improved at day 10, although the number of aberrant nuclei remained elevated up to day 30 after irradiation. When compared to sham irradiation (Figure 2A) the contact space between acini, but not between the cells within one acinus was reduced, suggesting interstitial oedema (Figure 2B). A slight elevation of fibrotic tissue was observed from day 60 on (not shown). In contrast to both the flow rate and the amylase secretion, the number of acinar cells recovered from day 120 postirradiation on (Figure 1B), accompanied by a second increase in aberrant nuclei (not shown) and in the number of vacuoles (not shown). Acini seemed to be enlarged and contained up to 30 acinar cells in a cross-section, compared to 10 in unirradiated acini (Figure 2C). The acinar cells within these acini, however, were disorganised and smaller. At 240 days after irradiation, this effect was more pronounced and much healthy tissue was replaced by fibrotic areas (Figure 2D).

Of the drugs used in this study, pilocarpine was of special interest, because it is already used in the clinic for the treatment of radiation therapy-induced xerostomia and salivary dysfunction (Johnson et al, 1993; Leveque et al, 1993; Valdez et al, 1993). It was shown earlier (Coppes et al, 1997b) that pilocarpine pretreatment in rats can reduce the acute and early effects of radiation on the parotid gland flow rate. This is confirmed here (Figure 3 (until day 60, $P < 0.05$, $n = 7$–8) ). Pilocarpine also reduces the acute and early reduction in amylase secretion (day 30–120, $P < 0.05$, $n = 6$–8 as well as the early decline in acinar cell number (day 30–60, $P < 0.01$, $n = 3$). The late effects, however, are not reduced (Figure 3). This urges the need for improved prophylactic treatments.

### Alternative drug pretreatments for early and late protection

In a previous study (Coppes et al, 1997a, b), we reported on a series of drug pretreatments that, like pilocarpine pretreatment, can attenuate the early radiation-induced decline in parotid gland function. As can be seen in Figure 4A, pretreatment with isoproterenol (ISO, up to day 120, $P < 0.05$, $n = 6$), methacholine (MCh, up to day 30, $P < 0.05$, $n = 7$), phenylephrine (PE) as well as a combination with PE and MCh indeed can attenuate the acute drop in saliva flow rate after irradiation. From day 10 on, however, the sparing effects of all pretreatments decreased up to day 60 (Figure 4B). From day 60 on the flow rates of the different experimental groups began to differ. Whereas the pilocarpine (Figure 3) and MCh pretreated groups no longer displayed a sparing effect on the flow rate, the sparing effects of PE, ISO and MCh + PE remained or even increased (Figure 4B). At 180 days after irradiation the ISO pretreated group no longer displayed sparing, whereas the PE ($P < 0.05$, $n = 6$) and especially MCh + PE ($P < 0.01$, $n = 6$) pretreated animals could uphold an increased saliva flow rate up to day 240 after irradiation, when compared to the untreated irradiated animals. So pretreatment with α-adrenergic receptor (α-AR) or α-AR + muscarinic acetylcholine receptor (MAChR) agonists led to long-term protection, whereas pretreatment with β-AR or MAChR agonists alone only transiently could ameliorate radiation-induced loss in flow rate.

All pretreatments reduced the effect of irradiation on amylase secretion (Figure 5). This effect was lost at day 240 after pretreatment with isoproterenol, pilocarpine and methacholine. However, similar to the flow rate pretreatment with PE/PE + MCh, being α-AR +/– MAChR agonists, lead to long-term sparing of amylase secretion ($P < 0.001$, $n = 5$ and $P < 0.001$, $n = 7$, respectively).

Neither in non-pretreated nor in drug pretreated rats significant cell loss was observed up to 10 days after irradiation. However, formation of vacuoles (Vissink et al, 1990; Peter et al, 1994a) was seen in non-pretreated but not in the drug pretreated rats (data not shown)

At day 30, the acinar cell number in the non-pretreated irradiated rats started to decline (Figure 1B, Figure 6). Except for methacholine plus phenylephrine, all pretreatments reduced the rate of cell loss ($P < 0.05$, $n = 3$).

The decline in cell number was less, whereas the numbers of aberrant nuclei were higher in drug-pretreated rats, when compared to the non-pretreated irradiated rats. This effect was most prominent after pilocarpine (Figure 3) and methacholine

 *© 2001 Cancer Research Campaign*

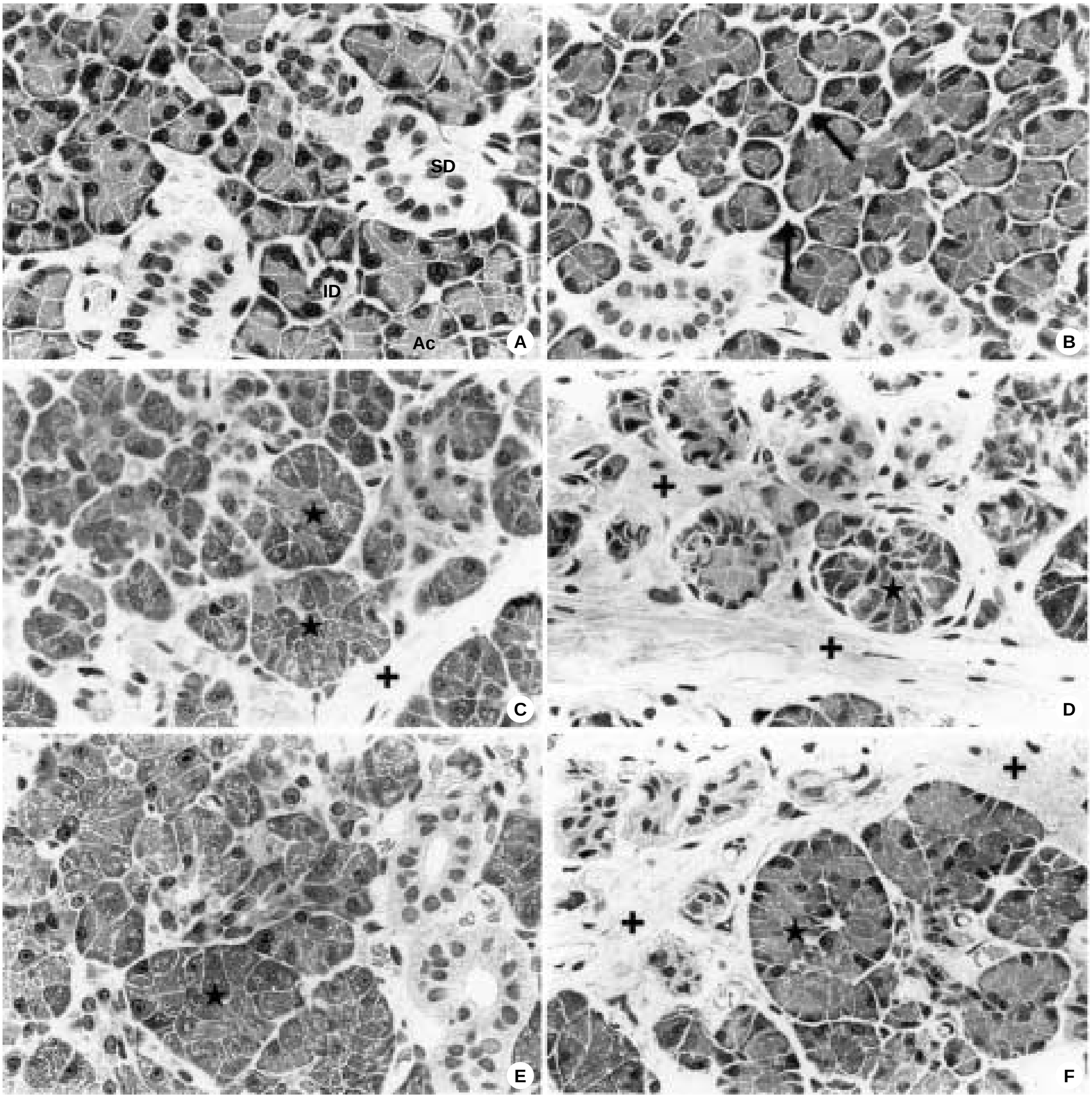

**Figure 2** Irradiation-induced damage to parotid gland tissue morphology. (**A**) Parotid gland from non-irradiation animals (Ac) acinar cells, (SD) striated duct cells and (ID) intercalated duct cells; (**B**) gland from a non-pretreated animal 10 days after irradiation with 15 Gy of X-rays: occurrence of interstitial oedema (→); (**C**) gland from a non-pretreated animal 120 days after 15 Gy of X-rays, showing large acini (*) and fibrosis (+); (**D**) gland from a non-pretreated animal 240 days after irradiation, showing large areas with fibrosis (+); (**E**) gland from a methacholine plus phenylephrine pretreated animal 240 days after irradiation, showing preserved general morphology with large acini (*); (**F**) gland from a pilocarpine-pre-treated animal 240 days after irradiation, showing a near to a similar picture as glands from irradiated non-pre-treated animals (**D**) with large acini (*) and fibrosis (+) (H&E, bars: 10 μm)

pretreatment but only marginal after methacholine plus phenylephrine pretreatment (Figure 6), possibly indicative of drug-induced delayed cell death. Beyond day 120 only in the methacholine plus phenylephrine pretreated group acinar cell numbers were still significantly higher than non-pretreated irradiated rats (Figure 6, $P < 0.001$, $n = 3$), whereas the number of aberrant nuclei were similar.

At day 240 after irradiation, the methacholine plus phenylephrine pretreated group displayed less fibrosis and a better but not normal structure of the acini than non-pretreated group (Figure 2E). The phenylephrine pretreated group showed intermediate morphological damage (not shown) and loss of cell number. All other drugs, including pilocarpine (Figure 2F) lost their protective effects both on number of cells as well as on morphology.

Table 1 summarises the effects of pretreatment with adrenergic and muscarinic agonists on the radiation-induced damage on flow rate. A composition is made from areas under the curve data, obtained from flow rate experiments from current (phenylephrine, isoproterenol, methacholine, pilocarpine and methacholine plus phenylephrine) and previous (methacholine plus isoproterenol and phenylephrine plus

© 2001 Cancer Research Campaign 

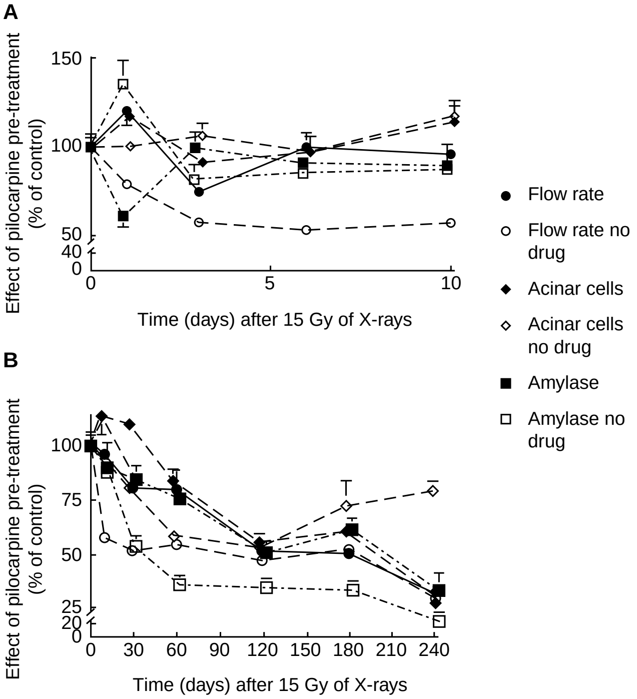

**Figure 3** Effects of pilocarpine pretreatment on changes in parotid gland flow rate (circles), amylase secretion (squares) and acinar cell number (diamonds) following local irradiation with 15 Gy of X-rays after pilocarpine pre-treatment. (**A**) Acute effects, 0–10 days after radiation. (**B**) Early to late effects, up to 240 days days after irradiation. Open symbols indicate the flow rates for non-pretreated animals following 15 Gy. The number of acinar cells are calculated as the number of cells counted multiplied by the ratio of the gland weight of the experimental rat and the average gland weight of age-matched control rats. Data are expressed as a percentage of pre-treatment control values and are the mean values (± SEM)

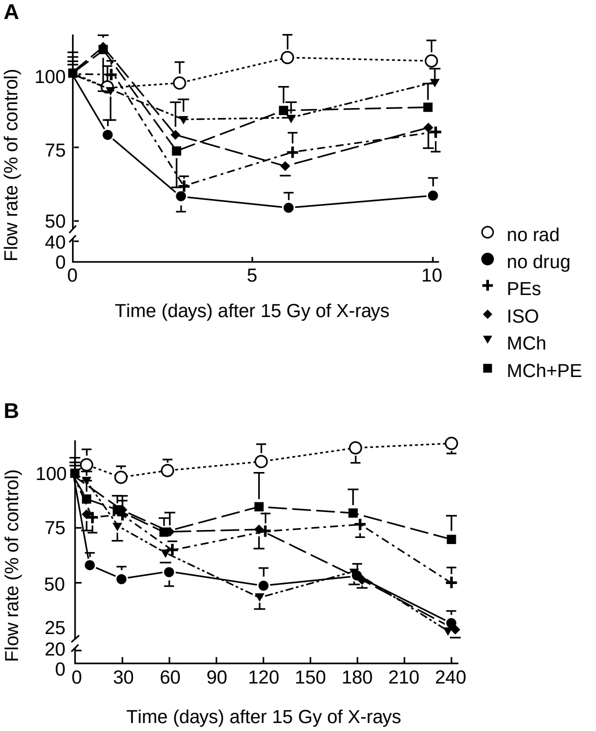

**Figure 4** Effect of drug pretreatments on changes in parotid gland flow rate following irradiation with 15 Gy of X-rays. (**A**) Acute effects, 0–10 days after radiation. (**B**) Early to late effects, up to 240 days after irradiation. No rad: Not pretreated, no irradiation; No drug: saline pre-treated. PE: phenylephrine, ISO: isoproterenol, MCh: methacholine, MChPE: methacholine plus phenylephrine. Data are expressed as a percentage of pretreatment control values and are the mean values (± SEM)

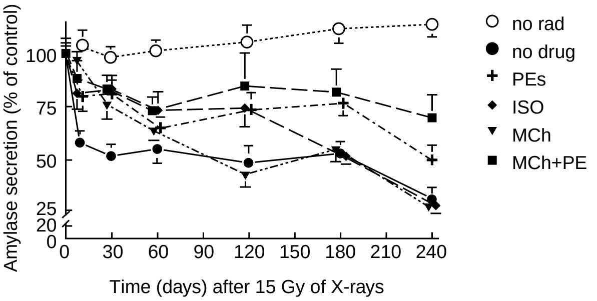

**Figure 5** Effect of drug pretreatments on changes in parotid gland amylase secretion following irradiation with 15 Gy of X-rays. No rad: not pre-treated, no irradiation; No drug: saline pre-treated. PE: phenylephrine, ISO: isoproterenol, MCh: methacholine, MChPE: methacholine plus phenylephrine. Data are expressed as a percentage of pre-treatment control values and are the mean values (± SEM)

isoproterenol (Coppes et al, 1997a) ) studies. All single applicated drugs attenuated the loss of salivary gland function after irradiation during the acute (0–10 days) and early (10–30 days) periods. The order of potency in which the different drugs spared saliva flow rate in the acute and early period is as follows: PILO ≥ MCh ≥ MCh + PE > ISO > PE. This indicates that stimulation of MAChRs prior to irradiation renders the best sparing properties during this time span. Combining MAChR + β-AR or α-AR + β-AR stimulation showed no protection at all. In the intermediate period (30–120 days) attenuation of salivary gland function by pilocarpine, methacholine and isoproterenol is almost lost. During the late period (120–240 days) only the combined methacholine plus phenylephrine and to a lesser degree phenylephrine alone uphold its sparing effect. During the late period the order of potency by the drugs used is as follows: MCh + PE > PE > ISO >> PILO = MCh. This indicates that for long-term sparing stimulation by α-AR agonists, especially combined with MAChR agonists renders the best sparing effects, whereas MAChR stimulation alone is ineffective.

### DISCUSSION

Salivary gland tissue is at risk during radiotherapy of head and neck cancer. The data presented here are the first to show the consecutive effects on radiation damage to the parotid gland and reveal that these effects may be divided into at least 4 distinct phases. These phases are differentially affected by the irradiation treatment. Also, the degree of sparing by pretreatments with muscarinic and/or adrenergic receptor agonists differs in extent and duration. Previously, it has been shown (Peter et al, 1995; Coppes et al, 1997a, b) that the early effects of radiation damage to these glands can be attenuated by pre-irradiation treatment with adrenergic and/or muscarinic receptor agonists. This includes the clinically applied drug pilocarpine. Our current data show that the latter drug is unable to protect against the long-term effects of a 15 Gy irradiation treatment when 100% of the parotid gland lies within the radiation portal. Most importantly, our results indicate a novel approach to ameliorate both early and late radiation effects on the parotid glands based on simultaneous pre-irradiation stimulation of muscarinic and α-adrenergic receptors. These effects were accomplished with non-clinically applicable drugs.

*© 2001 Cancer Research Campaign*

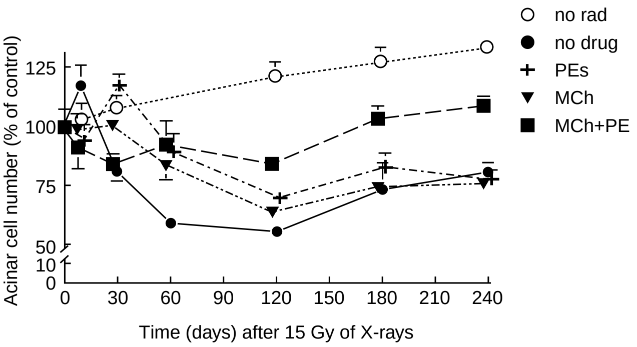

**Figure 6** Effect of drug pretreatments on changes in parotid gland acinar cell number following irradiation with 15 Gy of X-rays. No rad: not pre-treated, no irradiation; No drug: saline pre-treated. PE: phenylephrine, MCh: methacholine, MChPE: methacholine plus phenylephrine. The number of acinar cells are calculated as the number of cells counted multiplied by the ratio of the gland weight of the experimental rat and the average gland weight of age-matched control rats. Data are expressed as a percentage of pre-treatment control values and are the mean values (± SEM)

Therefore, knowledge on damage initiation and target cells, and there relation to effective drug pretreatments, might help to design clinically applicable long-term protectors of radiation damage to the parotid gland.

### Damage initiation and target cells

The degree of radiation damage inflicted to organs as well as the time of manifestation of the damage depend on the type and number of target cells involved. Saliva is firstly formed by acini as an isotonic primary secretion, which is then rendered hypotonic by the removal of sodium and chloride as it flows through the ductal system with little or no change in fluid volume (Garrett and Proctor, 1998). Therefore, acinar cells may be considered as primary target cells, which when damaged to their secretory potential or when disappeared (after cell killing), have an immediate effect on salivary secretion. Although not fully established, it seems (Budford-Mason et al, 1993; Redman, 1995; Denny et al, 1997; Dardick, 1998) that at least 2 types of acinar cells exist, cells capable of (some) division and end-stage cells. It is not clear whether or not these cells have equal secretion abilities. Radiation damage to acinar cells may therefore have different effects namely on secretion and on cell renewal. Dividing acinar cells may be considered primary as well as secondary target cells dependent on the type of damage manifested in these cells. Cell renewal, especially after irradiation (Peter et al, 1994b), may also be accomplished by division of intercalated duct cells. These cells may produce new acinar cells and as such be considered as tertiary target cells. In this view the primary target cells are responsible for the immediate loss of secretion capacity, the secondary target cells for impairment of acute cell renewal and the tertiary target cells for impairment of later cell renewal. How these target cells cope with the interaction with radiation depends, among other factors, on the physiological status of the cells. By pretreatment (e.g. by drugs) this status can be modified such that the cell is conditioned to better withstand the damaging action of radiation.

### Different phases of radiation damage and cell regeneration; effects of drug pretreatment

### *Acute damage (0–10 days)*

Interaction of radiation with parotid gland tissue immediately leads to an impaired saliva secretion, without loss of cell number. The primary target cells are damaged in their potential to secrete water but not in their potential to secrete amylase. The first mentioned cellular function is mainly connected with the activation of the $\alpha_1$-adrenergic receptor ($\alpha_1$-AR) and the $M_3$-muscarinic receptor ($M_3$-AchR) and the interaction with Gq-proteins (Sawaki et al, 1995), while secretion of proteins depends on β-adrenergic receptor (β-AR) activity and the interaction with Gs-proteins (Gomperts, 1990). We may not conclude, however, that radiation only damages the $\alpha_1$-AR and $M_3$-AChR signalling pathways and not the β-AR ones because it may very well be that overcapacity of the latter pathway compensates for the damage. Pretreatments with $\alpha_1$-AR, β-AR and $M_3$-AchR agonists protect the cells against function loss (Table 1). So the cells are conditioned by the pretreatment in such a way that they have become radioresistant. It is interesting to note that isoproterenol (β-AR agonist) protects against loss of $\alpha_1$-AR and $M_3$-AchR activities. Although not understood at the moment, it points to interactions between the different signalling pathways. The most interesting observation with respect to acute radiation damage is the phenomenon of non-protection when pretreatment with a β-AR agonist is coupled with pretreatment

**Table 1** Sparing effect by drug pre-treatment on the radiation induced damage to parotid gland flow rate. The follow up time is split into 4 periods: Acute (0–10 days), Early (10–60 days), Intermediate (60–120 days) and Late (120–240 days). The arrows indicate the sparing effect as derived from the flow rates experiments

| Drug | Receptor | Second messenger | Acute | Early | Intermediate | Late |
|---|---|---|---|---|---|---|
| Phenylephrine | $\alpha_1$-AR | $PLC/PIP_2$ | ↑ꜛ | ↑↑ | ↑ꜛ | ↑ |
| Isoproterenol | $\beta_1$-AR | AC/cAMP | ↑↑ | ↑↑ | ↑ꜛ | ○ |
| Pilocarpine | $M_3$-AchR | $PLC/PIP_2$ | ↑↑↑ꜛ | ↑↑ꜛ | ↑ | ○ |
| Methacholine | $M_3$-AchR | $PLC/PIP_2$ | ↑↑↑ | ↑↑ | ○ | ○ |
| Methacholine + Phenylephrine | $M_3$-AchR + $\alpha_1$-AR | $PLC/PIP_2$ $PLC/PIP_2$ | ↑↑ꜛ | ↑↑ꜛ | ↑↑ | ↑↑ |
| Methacholine + Isoproterenol | $M_3$-AchR + β-AR | $PLC/PIP_2$ AC/cAMP | ○ | ○ | nd | nd |
| Phenylephrine + Isoproterenol | $\alpha_1$-AR + β-AR | $PLC/PIP_2$ AC/cAMP | ○ | ○ | nd | nd |

○ no effect, ↑ and ꜛ about 25% and 12.5% protection, respectively, nd not determined.

with a $\alpha_1$-AR or $M_3$-AchR agonist (Coppes et al, 1997b, Table 1). This is likely due to mutual inhibition between the PLC/$PIP_2$ and AC/cAMP second messenger pathways (Fleming et al, 1992; Gerstin and Ehlert, 1996; Martinez and Zhang, 1998). When we accept the hypothesis of temporarily cell conditioning by the pretreatment then we have to conclude that the combined β-AR/$\alpha_1$-AR-$M_3$-AchR receptor activation prevents this metabolic conditioning. This cross-talk of signal transduction pathways in acinar cells is probably connected with phosphorylation reactions by cAMP/PKA on the muscarinic receptor-Gq protein system (Chang et al, 1996; Gerstin and Ehlert, 1996; Martinez and Zhang, 1998) and of PKC on the β-adrenergic-Gs protein system (Fleming et al, 1992; Chang et al, 1996). If this is true, cell conditioning leading to radioresistance might be caused by receptor activated (de)phosphorylation reactions on the coupled receptor–G protein system. So, acinar cells are considered as the primary target cells for acute radiation damage with the signal transduction pathways being the targets within these cells.

### *Early damage (10–60 days)*

While amylase secretion as well as the number of acinar cells did not change in the first period, both parameters decreased (Figure 1B) during the second period. The flow rate stayed constant at about 50% of the level of the non-irradiated gland. This suggests that between 10 and 60 days the acutely damaged cells were disappearing. There is no compensation of cells by dividing acinar cells, indicating radiation damage to the secondary targets too. All agents used protected (more or less to the same extent) against function loss during this period (Figure 2B, Table 1). The drugs used also protected against the reduction in amylase secretion and cell loss, although not all equally effective. This suggests that the drugs protect against both targets, being the receptor-effector signalling system leading to improved secretion and the molecular targets critical for improved cell survival and/or accelerated proliferation resulting in improved tissue renewal after irradiation.

### *Intermediate damage (60–120 days)*

In the period of 2 to 4 months after irradiation the damage expressed in terms of water and protein secretion as well as in terms of cell numbers is constant, when no pretreatment has taken place. Depending on the drugs used protection on flow rate is evident. The best protection (50%) is seen by the combination of methacholine plus phenylephrine. Methacholine alone does not protect at all. Furthermore, the protection against cell loss is much lower as seen from the combination with phenylephrine. Noteworthy is the relative low protection in this period by pilocarpine pretreatment. Differences between the protection against damage to the signalling pathway and the targets for cell killing become manifest.

### Late damage (120–240 days)

From a clinical perspective, this is the most important period studied. The amount of acinar cells increased (Figure 1B and Figure 6). Part of the increase may be explained by normal growth (see control, Figure 6) the other part may be considered as a response to radiation and the result of tissue replenishment via dividing acinar cells and cells of the intercalated duct. Despite the increase in cell number, the function of the gland deteriorates during this period to about 30% of the nonirradiated situation, which is consistent with previous observations by others (Funegard et al, 1997; Nagler et al, 1998). This probably relates to the abnormal morphology of the (large) acini observed at this time point (Figure 2D). The loss of flow rate can only be protected by pretreatment with phenylephrine but more effectively in combination with methacholine (Figure 4B, Table 1). The protection by these drugs is also evident for amylase secretion, number of cells, and tissue morphology. Our data could suggest that the combination of α-AR and MAChR stimulation by phenylephrine and methacholine, respectively, exerts an effect on the intercalated duct cells (tertiary target cells), necessary for long-term repopulation (Budford-Mason et al, 1993; Redman, 1995; Denny et al, 1997; Dardick, 1998).

Unfortunately, the drugs used in the current study cannot be used clinically in the concentration necessary to exert their effect. Yet, for the first time this study shows that the salivary gland can be protected against late radiation damage by pretreatment of receptors agonists, which have been shown to activate separately the PLC/PIP2 second messenger pathways (Sawaki et al, 1995), indicating possible involvement of these 2 second messenger pathways.

This finding opens the way for searching clinically applicable drugs that lead to cell conditioning by the same mechanism but with less toxic side effects as compared to the drugs used in the current study. This type of work is now ongoing in our laboratory.

In summary, the data show that radiation damage to the parotid gland can be described as 4 phases in time, firstly a rapid loss of function occurring without cell loss, followed by a phase of loss of acinar cells, hereafter a steady state phase, and finally a further deterioration of gland function and morphology, albeit with an increase in number of (abnormally arranged and probably nonfunctional) acinar cells. All drug pretreatments reduced the damage to the first 2 phases but only a combined stimulation of muscarinic acetylcholine receptors with methacholine plus α-adrenoceptors with phenylephrine can reduce both early and late damage.

### ACKNOWLEDGEMENTS

The authors wish to thank Dr AF Roffel and Dr A Vissink for valuable comments on the manuscript and Mr BFA Hellinga and Ing H Faber for the photographs. This work has been supported financially by grants of the Dutch Society for Cancer Research (GUKC 93-504 and RUG 98-1658).

### REFERENCES

Budford-Mason AP, Cummins MM, Brown DH, MacKay AJ and Dardick I (1993) Immunohistochemical analysis of the proliferative capacity of duct and acinar cells during ligation-induced atrophy and subsequent regeneration of the parotid gland. *J Oral Pathol Med* **22**: 440–446

Chang TT, Lacovelli L, Sallese M and De Blasi A (1996) G protein-coupled receptors: heterologous regulation of homologues desensitization and its implications. *Trends Pharmacol Sci* **17**: 416–421

Coppes RP, Zeilstra LJW, Vissink A and Konings AWT (1997a) Sialogogue-related radioprotection of salivary gland function: the degranulation concept revisited. *Radiat Res* **148**: 240–247

Coppes RP, Vissink A, Zeilstra LJW and Konings AWT (1997b) Muscarinic receptor stimulation increases tolerance of rat salivary gland function to radiation damage. *Int J Radiat Biol* **72**: 615–625

Dardick I (1998) Mounting evidence against current histogenetic concepts for salivary gland tumorigenesis. *Eur J Morphol* **36**: 257–261

Denny PC, Ball WD and Redman RS (1997) Salivary glands: a paradigm for diversity of gland development. *Crit Rev Oral Biol Med* **8**: 51–57

Eisbruch A, Haken RK, Hyungjin MK, Marsh LH and Ship JA (1999) Dose volume and function relationships in parotid salivary glands following conformal and intensity-modulated irradiation of head and neck cancer. *Int J Radiat Oncol Biol Phys* **45**: 577–587

 *© 2001 Cancer Research Campaign*

El Mofty SK and Kahn AJ (1981) Early membrane injury in lethally irradiated salivary gland cells. *Int J Radiat Biol* **9**: 55–62
Fleming N, Mellow L and Bhullar D (1992) Regulation of the cAMP signal transduction pathway by protein kinase C in rat submandibular cells. *Pflügers Arch* **421**: 82–89
Franzén L, Funegård U, Sundström S, Gustafsson H, Danielsson Å and Henriksson R (1991) Fractionated irradiation and early changes in salivary glands Different effects on potassium efflux exocytotic amylase release and gland morphology. *Lab Invest* **64**: 279–283
Funegard U, Ingegerd J, Franzen L, Ericson T, Nystrom H and Henriksson R (1997) Rat salivary gland function after fractionated irradiation. *Acta Oncol* **36**: 191–198
Garrett JR and Proctor GB (1998) Control of salivation In Front Oral Biol The Scientific Basis of Eating, Linden RWA (Ed) pp 135–155 Karger: Basel.
Gerstin EH and Ehlert JF (1996) Inhibition of muscarinic stimulated phosphoinositide hydrolysis in the rat parotid gland by Camp. *Life Sciences* **58**: 145–153
Gomperts BD (1996) A GTP-binding protein mediated exocytosis. *Ann Rev Physiol* **52**: 591–606
Gustafsson H, Franzén L, Sundström S and Henriksson R (1992) Different Effects of fractionated irradiation on potassium efflux and exocytotic amylase release. *Acta Otolaryngol* **492**: 94–98
Henricksson R, Fröjd Ö, Gustafsson H, Johansson S, Yi-Qing C, Franzén L and Bjermer L (1994) Increase in mast cells and hyaluronic acid correlates to radiation-induced damage and loss of serous acinar cells in salivary glands. The parotid and submandibular glands differ in radiation sensitivity. *Br J Cancer* **69**: 320–326
Jensen AB, Hansen O, Jørgensen K and Bastholt L (1994) Influence of late side-effects upon daily life after radiotherapy for laryngeal and pharyngeal cancer. *Acta Oncol* **33**: 487–491
Johnson JT, Ferretti GA, Nethery WJ, Valdez IH, Fox PC, Ng D, Muscoplat CC and Gallagher SC (1993) Oral pilocarpine for post-irradiation xerostomia in patients with head and neck cancer. *New Engl J Med* **329**: 390–395
Leslie MD and Dische S (1994) The early changes in salivary gland function during and after radiotherapy given for head and neck cancer. *Radiother Oncol* **30**: 26–32
Leveque FG, Montgomery M, Potter D, Zimmer MB, Ruiter JW, Steiger BW, Gallagher SC and Muscoplat CC (1993) A multicenter randomized double-blind placebo-controlled dose-titration study of oral pilocarpine for treatment of radiation-induced xerostomia in head and neck cancer patients. *J Clin Oncol* **11**: 1124–1131
Martinez JR and Zhang GH (1998) Cross-Talk in Signal Transduction Pathways of Rat Submandibular acinar cells. *Eur J Morph* **36**: 190–193
Nagler RM, Baum BJ and Fox PC (1993) Acute effects of X-irradiation on the function of rat salivary gland. *Radiat Res* **136**: 42–47
Nagler RM, Baum BJ, Miller G and Fox PC (1998) Long-term salivary effects of single-dose head and neck irradiation in the rat. *Arch Oral Biol* **43**: 297–303
Paardekooper GMRM, Cammelli S, Zeilstra LJW, Coppes RP and Konings AWT (1998) Radiation induced apoptosis is not the cause of acute impairment of rat salivary gland function. *Int J Radiat Biol* **73**: 641–648
Peter B, Van Waarde MAWH, Vissink A, ‘s-Gravenmade EJ and Konings AWT (1994a) Radiation-induced cell proliferation in the parotid and submandibular glands of the rat. *Radiat Res* **140**: 257–265
Peter B, Van Waarde MAWH, Vissink A, ‘s-Gravenmade EJ and Konings AWT (1994b) The role of secretory granules in radiosensitivity of salivary gland acini-A morphological study. *Radiat Res* **140**: 419–428
Peter B, van Waarde MAWH, Vissink A, ‘s Gravenmade EJ and Konings AWT (1995) The role of secretory granules in radiation-induced dysfunction of rat salivary glands. *Radiat Res* **141**: 176–182
Phillips RM (1970) X-ray-induced changes in function and structure of the parotid gland. *J Oral Surg* **28**: 432–437
Pierre KJ and Nadj H (1976) A new enzymatic kinetic method of determination of amylase. *Clin Chem* **22**: 12–19
Redman RS (1995) Proliferative activity by cell type in the developing rat parotid gland. *Anat Rec* **241**: 529–540
Roesink JM, Konings AW, Terhaard CH, Battermann JJ, Kampinga HH and Coppes RP (1999) Preservation of the rat parotid gland function after radiation by prophylactic pilocarpine treatment: radiation dose dependency and compensatory mechanisms. *Int J Radiat Oncol Biol Phys* **45**: 483–489
Sawaki K, Baum BJ and Ambudkar IS (1995) $\alpha_1$-Adrenergic and $m_3$-Muscarinic receptor stimulation of phosphatidylinositol 4,5-biphosphate-specific-phospholipase C are independently mediated by $G_{aq/11}$ in rat parotid gland membranes. *Arch Biochem Biophys* **316**: 535–540
Shannon IL, Trodahl JN and Starcke EN (1978) Radiosensitivity of the human parotid gland. *Proc Soc Exp Biol Med* **157**: 50–53
Sodicoff M, Pratt NE and Sholley MM (1974) Ultrastructural radiation injury of rat parotid gland: A histopathological dose response study. *Radiat Res* **58**: 196–208
United Kingdom Co-ordinating Committee on Cancer Research (UKCCCR) (1998) Guidelines for the Welfare of Animals in Experimental Neoplasia. *British Journal of Cancer* **77**(1): 1–10
Valdez IH, Atkinson JC, Ship JA and Fox PC (1992) Major salivary gland function in patients with radiation-induced xerostomia: flow rates and sialochemestry. *Int J Radiat Oncol Biol Phys* **25**: 41–47
Valdez IH, Wolff A, Atkinson JC, Macynski AA and Fox PC (1993) Use of pilocarpine during head and neck radiation therapy to reduce xerostomia and salivary dysfunction. *Cancer* **71**: 1848–1851
Vissink A, ‘s-Gravenmade EJ, Konings AWT and Ligeon EE (1989) The adaptation of the Lashley cup for use in rat saliva collection. *Arch Oral Biol* **34**: 577–578
Vissink A, ‘s-Gravenmade EJ, Ligeon EE and Konings AWT (1990) A functional and chemical study of radiation effects on rat parotid and submandibular/sublingual glands. *Radiat Res* **124**: 259–265
Vissink A, Kalicharan D, ‘s-Gravenmade EJ, Jongebloed WL, Ligeon EE, Nieuwenhuis P and Konings AWT (1991) Acute irradiation effects on morphology and function of rat submandibular glands. *Radiat Res* **124**: 259–265
Vissink A, Down JD and Konings AWT (1992) Contrasting dose rate effects of gamma-irradiation on rat salivary gland function. *Int J Radiat Biol* **61**: 275–282
Wolff A, Atkinson JC, Macynski AA and Fox PC (1990) Pretherapy interventions to modify salivary dysfunction. *NCI Monogr* **9**: 87–90
Zimmerman RP, Mark RJ, Tran LM and Juillard GF (1997) Concomitant pilocarpine during head and neck irradiation is associated with decreased posttreatment xerostomia. *Int J Radiat Oncol Biol Phys* **37**: 571–575

*© 2001 Cancer Research Campaign* 
